# Buffalo Medical and Surgical Association

**Published:** 1886-07

**Authors:** 


### Buffalo Medical and Surgical Association.

*Meeting of June 1, 1886.*

The President, Dr. W. W. Potter, in the chair; Dr. F. R. Campbell, Secretary. Present, Drs. Bartlett, Hayd, Grosvenor, Van Peyma, Burghardt, Strong, Wetmore, Crego, Howe, Hubbell, Eli Long, Brecht, Clark, Mann, Hartwig, Moody, Fowler, Park and Dorland.

Application for membership was made by Dr. E. G. Starr.

Dr. W. A. Wheeler was elected a member of the Association.

DR. W. W. POTTER read a paper, entitled,

SOME OBSERVATIONS ON THE USE OF THE UTERINE SOUND, AND ITS APPLICATION IN CLINICAL GYNÆCOLOGY.

(Published in this number of the JOURNAL.)

DISCUSSION.

DR. GROSVENOR did not entirely agree with the essayist. Had placed a great deal of confidence in the instrument, and considered it almost indispensable in some cases.

DR. HAYD, as one of the younger practitioners, thought that the sound should be used for making an accurate diagnosis. We should be willing to accept the responsibilities with the priviliges of an instrument. Had seen pelvic cellulitis follow its introduction.

DR. VAN PEYMA feared that the essayist had been too radical, and considered the sound a very valuable instrument.

DR. HARTWIG was pleased to hear an expert's opinion of the use of the sound. It has, undoubtedly, done more harm than good. Thought a bougie might be substituted for the sound in many cases. When the use of instruments became so general, the clinical history of cases were neglected. We can often make our diagnosis from history alone.

DR. STRONG thought that the sound could be used without danger, if ordinary care were observed. Was surprised to hear so many objections brought against it. Thought it would be exceedingly rash to dispense with its use entirely.

DR. WETMORE used the sound, principally, as a carrier of medicines. Thought it indispensable in diagnosing intra-uterine tumors.

DR. BARTLETT objected to the use of the sound for replacing mal-positions. Thought its chief use was to measure the depth of the uterine cavity. A good objection is the cloak it gives to the damnable practice of abortion. Thought that there was too much meddling with the interior of the womb. The curette was worse than the sound.

MRS. MOODY said she used the cylindrical speculum and sound daily, and had never done any harm with it. She preferred the silver probe as a carrier of medicines. She considered the sound useful in ascertaining the condition of the intra-uterine mucus membrane.

DR. MANN said that great harm was sometimes done by the careless use of the sound. Cited a case where the fundus of the uterus had been three times punctured in this way. He had the specimen in his possession. He also reported a case in which pelvic cellulitis followed the introduction of the sound, on four different occasions.

In conclusion, Dr. Potter stated that the discussion showed that they all held essentially the same views. He was opposed to the unnecessary, unskillful and careless use of the sound. He considered it unnecessary, as a rule, to ascertain the dimensions of the uterine cavity. The sound should never be passed through the round speculum.

*Voluntary Communications*—Dr. Howe gave a practical demonstration of the methods of experimenting with antiseptics and germicides, the cultivation of bacteria, and the construction and use of apparatus.

DR. PARK exhibited the apparatus employed in the intubation of the larynx.

*Reports of Committees.*—Dr. W. W. Potter, as Chairman of the Committee on Rooms, reported that the Association would hold its meetings in the "Genesee," until permanent quarters could be provided.

The Treasurer, Dr. Brecht, reported that there was no record of the election of Drs. J. W. Putnam, Hinkel, Bode and Niemand.

The Secretary was instructed to cast the ballot of the Association for their election. The President declared Drs. Putnam, Hinkel, Bode and Niemand duly elected members of the Association.

*Prevailing Disease.*—Whooping cough.

The President announced that Dr. Dorland would be the essayist at the July meeting. Subject—" Hysteria."

The meeting then adjourned.

